# The effect of osteoporotic and non-osteoporotic individuals’ T cell-derived exosomes on osteoblast cells’ bone remodeling related genes expression and alkaline phosphatase activity

**DOI:** 10.1186/s13104-022-06139-4

**Published:** 2022-08-08

**Authors:** Mohammad Hasan Omidvar, Mohammad Sadegh Soltani-Zangbar, Majid Zamani, Roza Motavalli, Mehdi Jafarpoor, Sanam Dolati, Majid Ahmadi, Amir Mehdizadeh, Alireza Khabbazi, Mehrzad Hajialilo, Mehdi Yousefi

**Affiliations:** 1 Connective Tissue Diseases Research Center, Tabriz University of Medical Sciences, Tabriz, Iran; 2 Student Research Committee, Tabriz University of Medical Sciences, Tabriz, Iran; 3 Stem Cell Research Center, Tabriz University of Medical Sciences, Tabriz, Iran; 4 Department of Immunology, School of Medicine, Tabriz University of Medical Sciences, Tabriz, Iran; 5 Department of Medical Laboratory Sciences, Faculty of Allied Medicine, Infectious Diseases Research Center, Gonabad University of Medical Sciences, Gonabad, Iran; 6 Physical Medicine and Rehabilitation Research Center, Aging Research Institute, Tabriz University of Medical Sciences, Tabriz, Iran; 7 Hematology and Oncology Research Center, Tabriz University of Medical Sciences, Tabriz, Iran

**Keywords:** Osteoporosis, Osteoimmunology, Exosomes, Type I collagen, Osteopontin, Osteocalcin, Alkaline phosphatase

## Abstract

**Objectives:**

Osteoporosis is a common skeletal disorder attributed to age and is defined as a systematic degradation of bone mass and the microarchitecture leading to bone fractures. Exosomes have been reported in almost all biological fluids and during the failure of bone remodeling. 20 ml of blood samples were obtained from osteoporotic and non-osteoporotic postmenopausal women. After the isolation of peripheral blood mononuclear cells (PBMCs), T cells were separated via the magnetic-activated cell sorting (MACS) technique. Exosomes were driven from T cells of non-osteoporotic and osteoporotic volunteers. Subsequently, normal osteoblasts were treated with obtained T cell exosomes to assess osteoblastic function and gene expression.

**Results:**

Runx2, type I collagen, osteopontin, and osteocalcin expression decreased in osteoblasts treated by osteoporotic T cell exosomes. In contrast, an increased expression of the mentioned genes was observed following non-osteoporotic T cell exosome treatment. Additionally, osteoblast alkaline phosphatase (ALP) activity treated with non-osteoporotic T cell exosomes increased. However, this activity decreased in another group. Our data demonstrated that T cell exosomes obtained from osteoporotic and non-osteoporotic individuals could alter the osteoblastic function and gene expression by affecting the genes essential for bone remodeling.

**Supplementary Information:**

The online version contains supplementary material available at 10.1186/s13104-022-06139-4.

## Introduction

Osteoporosis is characterized as a systemic skeletal age-related condition with low bone mass and microarchitectural degradation of bone tissue, leading to elevated rates of bone fragility and vulnerability to fractures [1, 2].

The critical function of osteoblasts is to synthesize high-collagen organic materials by secreting various matrix metalloproteinase (MMP) and bone matrix proteins to provide ideal conditions for matrix mineralization [3]. Mature osteoblasts can continue mineralization by expressing alkaline phosphatase (ALP), osteopontin, and osteocalcin [4]. ALP may be found in a wide range of species, from bacteria to humans. The enzymes catalyze the hydrolysis of phosphoric acid monoesters as well as a transphosphorylation process in the presence of high phosphate acceptor concentrations [5]. In metabolic bone illnesses such as osteoporosis, osteomalacia, and rickets, as well as hyperparathyroidism, renal osteodystrophy, and thyrotoxicosis, ALP levels rise [6]. Three transcription factors encoded by the mammalian runt-related gene family serve critical roles in lineage-specific cell proliferation and differentiation. The master transcription factor Runt-related transcription factor 2 (Runx2) controls osteoblast differentiation [7]. The Runx2 gene is expressed early in osteoblastic development and is up-regulated in proliferative chondrocytes. Multiple signal transduction pathways can activate this gene and directly activate the transcription of osteoblast-related genes such as osteocalcin, osteopontin, and type I collagen [8]. In this regard, it has been demonstrated that the Runx2 null mouse (129/Sv strain) displays a total loss of the bone [9].

The field of osteoimmunology is an interdisciplinary area of study that focuses on the molecular interpretation of immune-skeletal interactions [10]. Osteoclastogenesis is mainly controlled by the nuclear factor-κB ligand-receptor activator (RANKL), which is the main culprit for improved osteoclastic activation. RANKL is a type II membrane protein that belongs to the TNF superfamily and has a transmembrane domain and a C-terminal receptor-binding domain. RANKL is a critical predictor of the degree of bone resorption in vivo and is expressed by osteoclastogenesis-supporting cells, including osteoblasts, in response to osteoclastogenic stimuli, including prostaglandin E2 (PGE2), 1,25-dihydroxyvitamin D3 (1,25 (OH) 2D3), and parathyroid hormone [11]. The expression of RANKL could also be directly or indirectly increased by activated T cells, which facilitate osteoclastic activity [12, 13].

Exosomes are membranous vesicles with a size of 40–150 nm in diameter secreted by most cells and are integral components of the microenvironment [14–17]. Throughout bone remodeling failures, osteoblastic or osteoclastic networks could be regulated by exosomes [18, 19]. On the other hand, molecular modifications in serum-derived exosomes (SDEs) of elderly individuals with reduced bone mass and bone remodeling functions remain thoroughly elucidated. Herein, we hypothesized that T-cell exosomes of postmenopausal women, with or without osteoporotic diseases, could alter normal osteoblasts function and molecular mechanisms such as Runx2, type I collagen, osteopontin, osteocalcin, and ALP activity.

## Main text

### Materials and methods

#### Study design

Postmenopausal participants were divided into non-osteoporotic (N = 21) and osteoporotic (N = 25) groups based on Bone mineral density (BMD) T scores in accordance with the World Health Organization osteoporosis characterization. BMD measurement was performed for the entire individuals via Dual-energy x-ray absorptiometry (DEXA). None of these volunteers had received chemotherapy/radiation treatment before blood sampling. Alcohol consumption, steroid use, hospitalization, previous fractures, kidney diseases, or cancer were also considered exclusion criteria.

#### T-cell isolation

From osteoporotic and non-osteoporotic postmenopausal women, 20 ml of peripheral blood was obtained sterilely. PBMCs were isolated from samples using Ficoll (lymphosep) 1.077 g/ml (Biosera Inc., East Sussex, UK) and gradient centrifugation 25 min, 450*g* technique. Magnetic-activated cell sorting (MACS) technique was also used to isolate T cells with a negative selection protocol using the Pan T Cell Isolation Kit (Order no. 130-096-535; Miltenyi Biotec, San Diego), as recommended by the manufacturer.

#### Exosome isolation

T-cell samples were centrifuged for 10 min at 1500*g*. Then, the obtained supernatant was centrifuged for 15 min at 17,000*g*, after which the supernatant was spun again for 1 h at 160,000g by an ultracentrifuge. Using the western blotting technique, the exosomes in the obtained pellet were identified by exosomal markers, including CD81, CD63, and CD9. Also, the morphology and size of the exosomes were evaluated by scanning electron microscope (SEM).

#### Cell culture

Isolation of Human osteoblasts (HOBs) was done from femoral heads of patients undergoing hip replacement surgery, as described previously [20]. Briefly, isolated bone samples were refined from the soft tissue and broken down into small fragments. Digestion was performed three times in a mixture of 2.0 mg/ml collagenase P (Roche Diagnostics, Germany) and 0.7 mg/ml collagenase II (Biochrom Germany), both from clostridium histolyticum, dissolved in phosphate‐buffered saline (PBS; PAA Laboratories, Austria) with 30 min of gentle agitation at 37 °C. Then, bone fragments culture was performed at 37°C in a water-saturated atmosphere with 5% CO2 in α-minimum essential medium (α-MEM) supplemented with 10% fetal bovine serum, 100 μg/ml streptomycin, and 100 IU/ml penicillin (Invitrogen Germany). After 3–4 weeks, the cells were trypsinized, transferred to plates, and cultured using the same α-MEM medium as explained above. These cells were treated with T cells exosomes driven from non-osteoporotic and osteoporotic volunteers.

#### Real-time quantitative PCR

Then, bone fragments culture was performed at 37 °C in a water-saturated atmosphere with 5% CO2 in α-minimum essential medium (α-MEM) supplemented with 10% fetal bovine serum, 100 μg/ml streptomycin, and 100 IU/ml penicillin (Invitrogen Germany). After 3–4 weeks, the cells were trypsinized, transferred to plates, and cultured using the same α-MEM medium as explained above. These cells were treated with T cells exosomes driven from non-osteoporotic and osteoporotic volunteers.

#### ALP activity

ALP activity was colorimetrically assessed by employing para nitrophenyl phosphate as the substrate (ALP kit 104-LL; Sigma), described previously by Kura et al. [21]. Briefly, cells were seeded in a density of 100 cells/well in 96-well plate format and incubated in a complete culture medium containing obtained exosomes from ordinary or osteoporotic postmenopausal women. Wells without exosomes and enjoying merely culture medium were considered as controls. After 14 days of incubation, cells were washed twice with PBS and subsequently solubilized with 1% Triton-X (BDH Laboratory Supplies, Poole, UK) (50 μL/well) for 20 min. Then, 50 μL of 1.5 mM 2-amino-2- methyl-1-propanol (pH 10.3) (Sigma) and four mM para nitrophenyl phosphate disodium (Sigma) mixture was added to each well and incubated at 37 °C for 30 min. The reaction was terminated by adding 150 μL of 1 M NaOH. ALP activity determination was performed by measuring the optical density at 405 nm (A405) via a spectrophotometer (Titertek Multiskan Plus, Helsinki, Finland). The standard curve of the Sigma Units was used to calculate the experimental samples' International Units (IU/L).

#### Statistical analysis

Data were presented as mean ± standard deviation (SD) of triplicate experiments. The statistical differences of this cross-sectional study were analyzed via one-way ANOVA analysis of variance, as well as a post hoc test (Dunnett’s T3 multiple comparisons test) for determining group differences in study parameters, and p < 0.05 was regarded as statistically significant. SPSS software (version 24.0 for Windows; Armonk, NY, USA) and Prism software (GraphPad Prism for Windows, version 6.01; Nashville, TN, USA) were employed to implement the fundamental statistical analyses.

## Results

### General characteristics of individuals

Supplementary Table 1: Additional file 2 shows the general characteristics of the individuals. Significant differences were observed regarding the lumbar T-score (−0.46 ± 0.45 versus −2.57 ± 0.82, p = 0.0001), femur T-score (0.32±0.09 versus −1.76 ± 0.65, p = 0.0001), total lumbar BMD (1.047 ± 0.074 versus 0.726 ± 0.083, p = 0.0001) and total femur BMD (1.114 ± 0.063 versus 0.813 ± 0.059, p = 0.0001) between mentioned groups, respectively.

### Isolated T-cell exosomes identification

SEM microscopy was used to show isolated exosome morphology (Additional file 1: Fig. S1-A). Western blot was performed to detect the expressions of specific surface markers of exosomes, and the results confirmed the presence of CD9, CD63, and CD81. Positive and negative controls were used to validate western blotting results (Additional file 1: Fig. S1–B).

### Gene expression

**Fig. 1:**
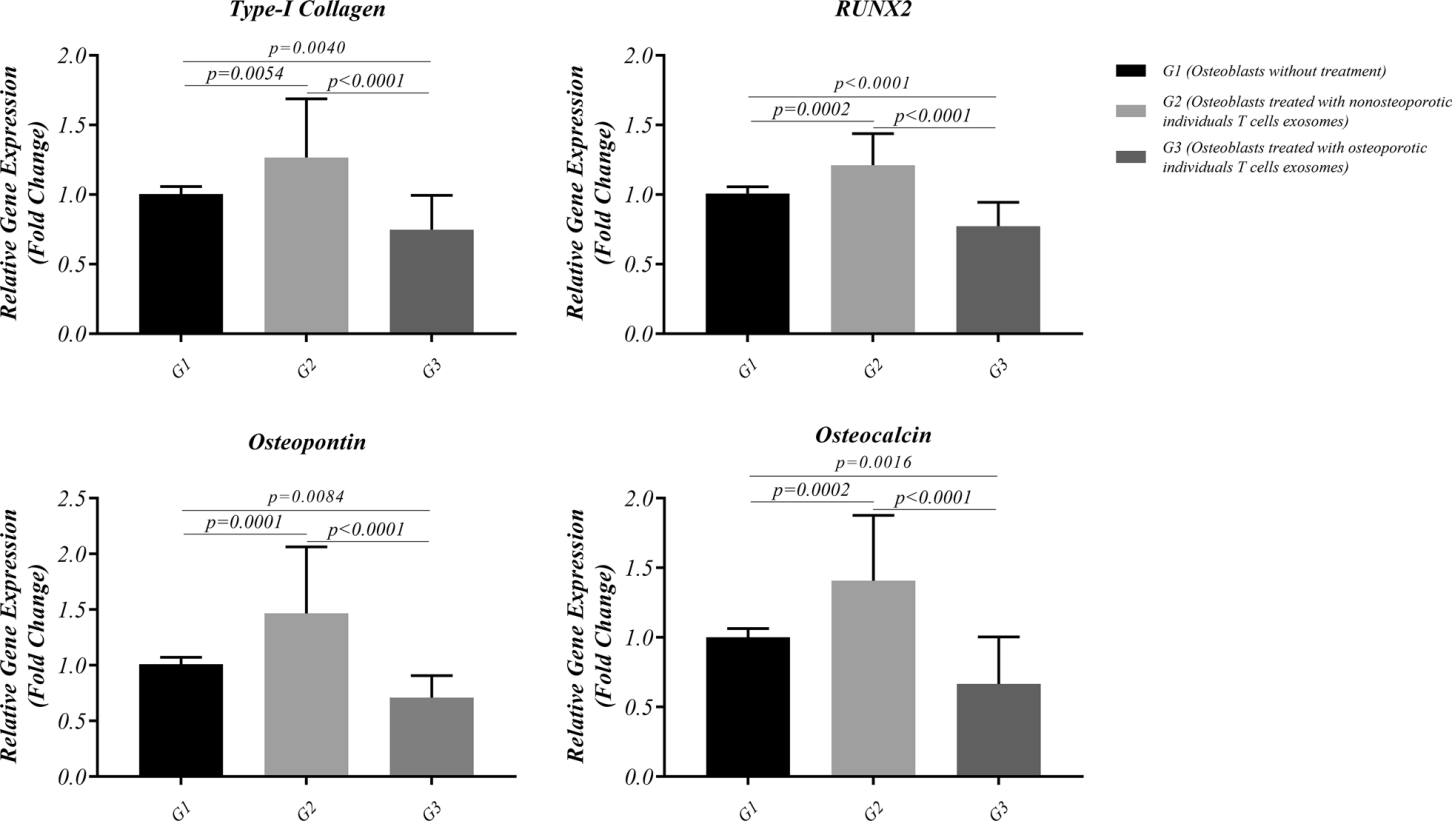
The expression level of type I collagen, RUNX2, Osteopontin, and osteocalcin after treatment with T cell exosomes from postmenopausal women with or without osteoporosis. The expression level of these genes was amplified after treatment of exosomal T-cells driven by non-osteoporotic individuals. In contrast, the exposure to T-cell exosomes from osteoporotic people deteriorated gene expression levels. Data are presented as mean±SD. P < 0.05 was considered statistically significant

Table 1 and Fig. 1 show the gene expression profile of normal osteoblasts after treatment with T cell exosomes. G1, G2, and G3 represent controls, osteoblasts treated with non-osteoporotic T cells exosomes, and osteoblasts treated with osteoporotic T cells exosomes, respectively. Required primer pairs for evaluation of related genes are provided in Additional file 3: Table S2. A significant increased Runx2 (1.211 ± 0.2259 versus 1.007 ± 0.04826, p = 0.0002 and 0.772 ± 0.1727, p < 0.0001, respectively), type I collagen (1.266 ± 0.4215 versus 1.005 ± 0.05339, p = 0.0054 and 0.7472 ± 0.2469, p<0.0001, respectively), osteopontin (1.465 ± 0.5981 versus 1.01 ± 0.06045, p = 0.0001 and 0.7084 ± 0.1989, p < 0.0001, respectively), and osteocalcin (1.408 ± 0.4706 versus 1.000 ± 0.06301, p = 0.0002 and 0.666 ± 0.3378, p < 0.0001, respectively) expression was observed in G2 compared to G1 and G3. The expression level of mentioned genes was increased by treatment of exosomal T-cell driven from non-osteoporotic individuals, while exposure to T-cell exosomes from osteoporotic persons declined gene expression level. Meanwhile, the expression levels of mentioned genes considerably decreased in G3 as compared to G1 and G2. Meanwhile, the gene expression levels considerably decreased in G3 compared to G1 and G2. Alkaline phosphatase gene expression results showed a significant increase when osteoblasts were treated with non-osteoporotic T cells exosomes compared to osteoblasts treated with osteoporotic T cells exosomes (1.276 ± 0.4766 versus 0.9390 ± 0.2215, p  =  0.0205).

**Table 1:** Osteoblasts gene expression and alkaline phosphatase activity after treatment with non-osteoporotic and osteoporotic T cells exosomes

Target	G1 mean±SD (n = 25)	G2 mean±SD (n = 21)	G3 mean±SD (n = 25)	P value		
				G1 vs G2	G1 vs G3	G2 vs G3
Gene expression level						
RUNX2	1.007±0.04826	1.211±0.2259	0.772±0.1727	0.0002	<0.0001	<0.0001
Type-I collagen	1.005±0.05339	1.266±0.4215	0.7472±0.2469	0.0054	0.0040	<0.0001
Osteopontin	1.01±0.06045	1.465±0.5981	0.7084±0.1989	0.0001	0.0084	<0.0001
Osteocalcin	1.000±0.06301	1.408±0.4706	0.666±0.3378	0.0002	0.0016	<0.0001
ALP	1.000±0.08517	1.276±0.4766	0.9390±0.2215	NS	NS	0.0205
Alkaline phosphatase (ALP) activity						
ALP (U/L)	77.56±20.08	93.68±30.30	70.10±18.71	NS	NS	0.0039

Data are presented as mean±SD

*G1* osteoblasts without treatment, *G2* osteoblasts treated with non-osteoporotic individuals T cells exosomes, *G3* osteoblasts treated with osteoporotic individuals T cells exosomes, *ALP* alkaline phosphatase

p<0.05 was considered as statistically significant

### ALP activity

**Fig. 2:**
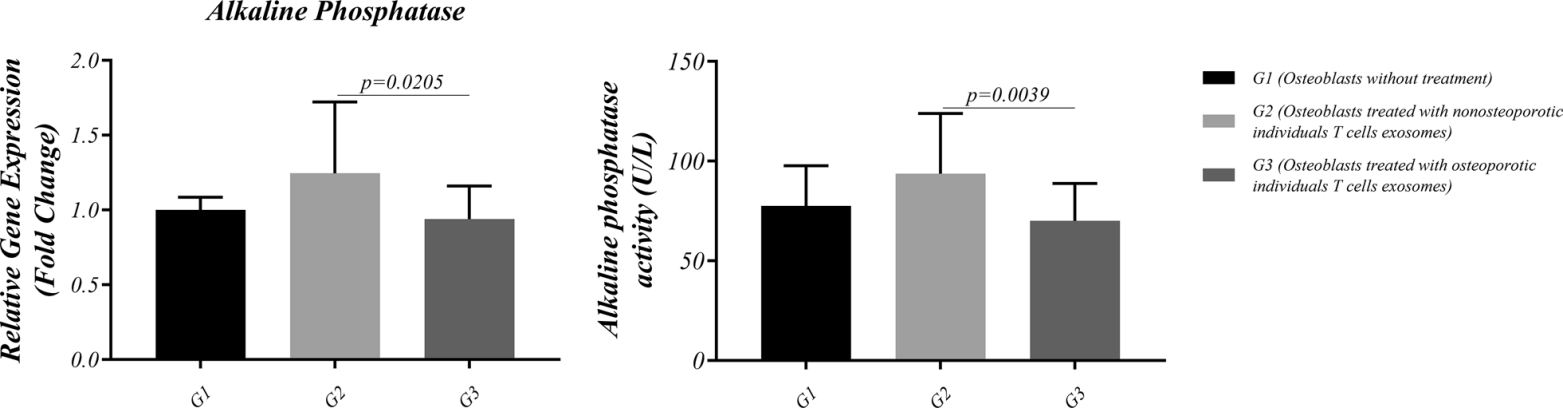
Alkaline phosphatase gene expression and activity after treatment with different T cell exosomes. The osteoblasts’ ALP activity increased after non-osteoporotic T-cell exosome treatment. However, the activity of ALP in osteoblasts decreased by exosomal T-cell treatment obtained from osteoporotic individuals. Data are presented as mean±SD. P < 0.05 was considered statistically significant

After osteoblast cells were treated with different T cell exosomes, ALP activity was assessed (Table 1, Fig. 2). Data revealed a significant ALP activity in G3 compared to G2 (70.10 ± 18.71 versus 93.68 ± 30.30, p = 0.0039). The results show that osteoblast’s ALP activity decreased after osteoporotic T-cell exosome exposure, though this activity surged by exosomal T-cell treatment obtained from non-osteoporotic individuals. However, no significant difference was observed between G1 and G2 or G1 and G3.

## Discussion

The central regulator of osteoclastogenesis is RANKL, which triggers this process and immensely contributes to a mechanism known as bone remodeling [22, 23]. Conversely, Runx2 and downstream molecules are critical for osteoblastic differentiation and lineage commitment [24, 25]. Herein, we demonstrated that exosomes driven from T cells could change the gene expression Runx2, type I collagen, osteopontin, and osteocalcin. In this regard, the expression level of Runx2 in osteoblasts treated with non-osteoporotic T cell exosomes considerably increased. In contrast, the expression level of Runx2 in osteoblasts treated with T cell exosomes of osteoporotic persons significantly declined. Serum osteocalcin level is considered a sensitive bone marker related to high bone turnover rates and BMD reduction and is well linked to histomorphometric bone formation [26]. Osteopontin is linked to bone resistance and bone remodeling [27]. Our results revealed that T cell exosomes driven from osteoporotic individuals could deleteriously affect Runx2 functions by osteoblast-related gene downregulation, including osteocalcin, type I collagen, and osteopontin. On the other hand, treatment via non-osteoporotic T cell exosomes increased the expression levels of these genes in osteoblasts [28].

This research observed increased ALP activity in osteoblasts treated with non-osteoporotic T cell exosomes compared to the group treated with osteoporotic T cell exosomes. Piatelly et al. [29] tried to show bone formation around dental implants used in conjunction with ALP extracted from the calf intestine. Their results suggested that ALP seems, in the experimental conditions, to have a positive effect on bone formation around titanium plasma-sprayed implants. In another study, Stucki et al. [30] showed that alkaline phosphatase activity is vital in the early stages of guided bone regeneration.

In vitro research showed that monocyte exosomes stimulate osteogenic differentiation in mesenchymal stem cells (MSCs) [31]. Rux2 plays a vital role in osteogenic differentiation and induces MSCs differentiation into pre-osteoblasts [32]. Our data showed that treating osteoblasts T cell exosomes obtained from osteoporotic persons could downregulate the expression level of Runx2, which is essential for osteoblast differentiation. However, Runx2 expression was increased in osteoblasts treated with non-osteoporotic T cell exosomes.

## Conclusion

Overall, T cell exosomes obtained from osteoporotic patients could alter osteoblastic function (ALP activity) and gene expression through downregulation of essential genes for bone formation such as Runx2, type I collagen, osteopontin, and osteocalcin, consequently propelling cells towards osteoclastogenesis. Meanwhile, treating osteoblasts with non-osteoporotic T cell exosomes could up-regulate bone formation by increasing the expression level of mentioned genes responsible for bone construction. Exosomes provide an excellent therapeutic opportunity and might be considered a trustworthy and novel solution for the treatment of osteoporosis due to their many inherent benefits, including low toxicity and immunogenicity.

## Limitations

A limitation of our study with as the small sample size.

## Supplementary Information

**Additional file 1: Figure S1.** Exosomes were observed by SEM images of exosomes isolated from T Lymphocytes. They showed the spherical and cup-shaped morphology of MVs ranging in size to 50 nm. (B) Western blotting analysis of CD9, CD63, and CD81 as exosome surface markers.

**Additional file 2: Table S1**. General characteristics of non-osteoporotic and osteoporotic postmenopausal volunteers.

**Additional file 3: Table S2.** Primer sequences of evaluated genes.

## Data Availability

All the necessary data are presented herewith. However, if needed, raw data in excel format can be availed on reasonable request from the corresponding author.

## Abbreviations

ALP: Alkaline phosphatase
BMD: Bone mineral density
BMPs: Bone morphogenetic proteins
DEXA: Dual-energy x-ray absorptiometry
M-CSF: Macrophage colony-stimulating factor
MMP: Matrix metalloproteinase
MSCs: Mesenchymal stem cells
OPG: Osteoprotegerin
PBMCs: Peripheral blood mononuclear cells
RANKL: Receptor activator of nuclear factor-κB ligand
Runx2: Runt-related transcription factor 2
SDEs: Serum-derived exosomes
TNF: Tumor Factor Necrosis factor
