# Supplementary figures and images for: The effect of osteoporotic and non-osteoporotic individuals’ T cell-derived exosomes on osteoblast cells’ bone remodeling related genes expression and alkaline phosphatase activity
Source: BMC Res Notes. 2022 Aug 8;15:272. doi: 10.1186/s13104-022-06139-4 (PMC9358836; doi:10.1186/s13104-022-06139-4)

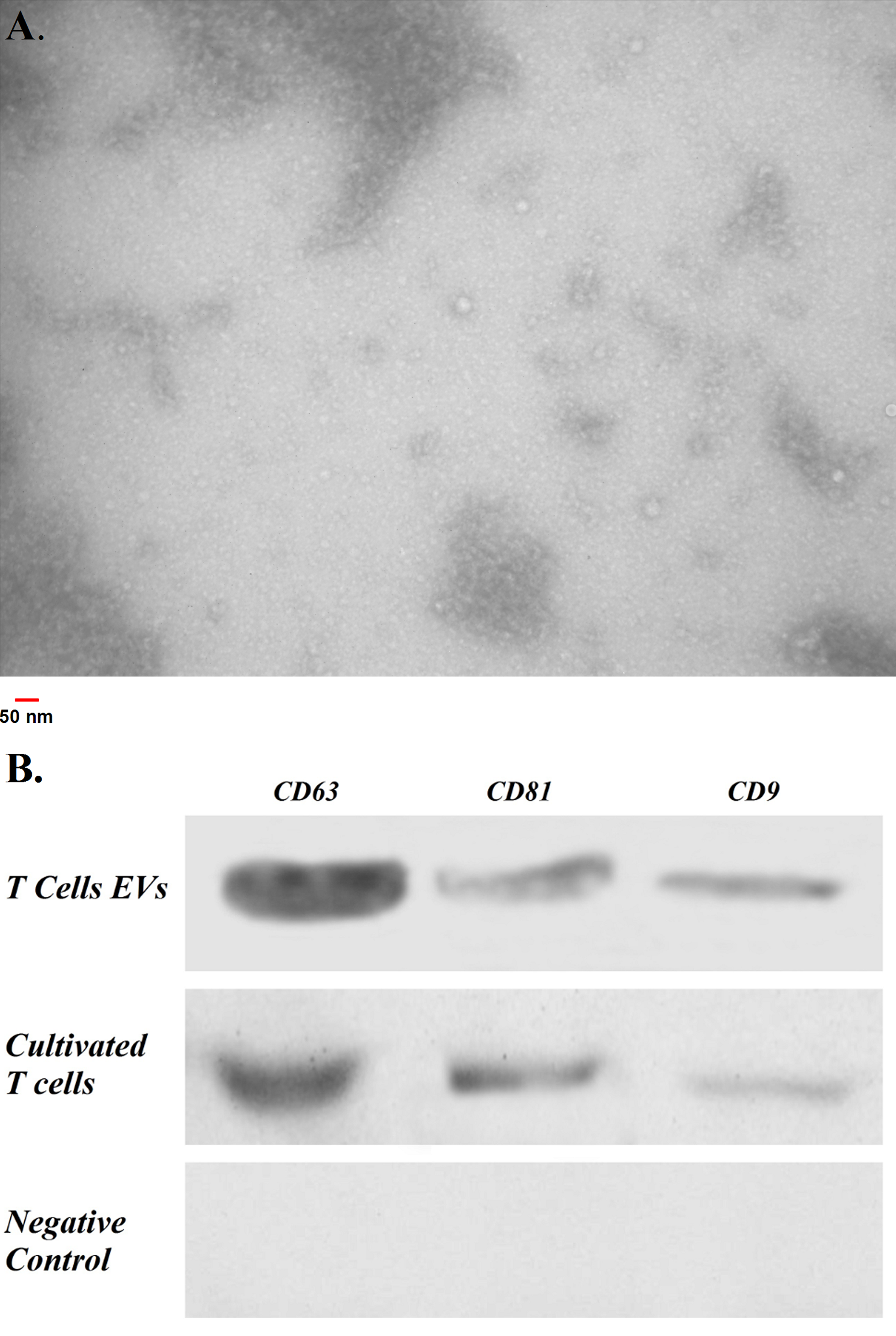

Supplement: Supplementary file 1 — Additional file 1: Figure S1. Exosomes were observed by SEM images of exosomes isolated from T Lymphocytes. They showed the spherical and cup-shaped morphology of MVs ranging in size to 50 nm. (B) Western blotting analysis of CD9, CD63, and CD81 as exosome surface markers. [file 13104_2022_6139_MOESM1_ESM.tif]
