# Supplementary material for: The effect of osteoporotic and non-osteoporotic individuals’ T cell-derived exosomes on osteoblast cells’ bone remodeling related genes expression and alkaline phosphatase activity
Source: BMC Res Notes. 2022 Aug 8;15:272. doi: 10.1186/s13104-022-06139-4 (PMC9358836; doi:10.1186/s13104-022-06139-4)
Supplement: Supplementary file 2 — Additional file 2: Table S1. General characteristics of non-osteoporotic and osteoporotic postmenopausal volunteers. [file 13104_2022_6139_MOESM2_ESM.docx]

**Supplementary Table 1**. General characteristics of non-osteoporotic and osteoporotic postmenopausal volunteers.

| Variables | Postmenopausal  Non-osteoporotic | Postmenopausal  osteoporotic | P value |
| --- | --- | --- | --- |
| Number | 21 | 25 | - |
| Age(years) | 54.23±5.94 | 61.43±7.09 | 0.059 |
| Weight (kg) | 67.33±10.93 | 61.55±8.80 | 0.035 |
| Height (m) | 1.62±0.095 | 1.61±0.05 | 0.82 |
| BMI (kg/m2) | 28.99±4.65 | 27.88±3.45 | 0.77 |
| Lumbar T-score | -0.46±0.45 | -2.57±0.82 | 0.0001 |
| Femur T-score | 0.32±0.09 | -1.76±0.65 | 0.0001 |
| Total lumbar BMD | 1.047±0.074 | 0.726±0.083 | 0.0001 |
| Total femur BMD | 1.114±0.063 | 0.813±0.059 | 0.0001 |

Data are presented as mean±SD. p<0.05 was considered as statistically significant.
