# Supplementary material for: The effect of osteoporotic and non-osteoporotic individuals’ T cell-derived exosomes on osteoblast cells’ bone remodeling related genes expression and alkaline phosphatase activity
Source: BMC Res Notes. 2022 Aug 8;15:272. doi: 10.1186/s13104-022-06139-4 (PMC9358836; doi:10.1186/s13104-022-06139-4)
Supplement: Supplementary file 3 — Additional file 3: Table S2. Primer sequences of evaluated genes. [file 13104_2022_6139_MOESM3_ESM.docx]

**Supplementary Table 2:** Primer sequences of evaluated genes

| **Gene** | **Primer** | **Sequence (5′→3′)** |
| --- | --- | --- |
| *Type-I Collagen* | Forward  Reverse | GATTCCCTGGACCTAAAGGTGC  AGCCTCTCCATCTTTGCCAGCA |
| *RUNX2* | Forward  Reverse | CCCAGTATGAGAGTAGGTGTCC  GGGTAAGACTGGTCATAGGACC |
| *Osteopontin* | Forward  Reverse | CGAGGTGATAGTGTGGTTTATGG  GCACCATTCAACTCCTCGCTTTC |
| *Osteocalcin* | Forward  Reverse | CGCTACCTGTATCAATGGCTGG  CTCCTGAAAGCCGATGTGGTCA |
| *Alkaline Phosphatase* | Forward  Reverse | GCTGTAAGGACATCGCCTACCA  CCTGGCTTTCTCGTCACTCTCA |
| *GAPDH* | Forward  Reverse | GTCTCCTCTGACTTCAACAGCG  ACCACCCTGTTGCTGTAGCCAA |
